# Crystal structure of 5-(4-meth­oxy­phen­yl)-3-(4-methyl­phen­yl)-4,5-di­hydro-1*H*-pyrazole-1-carbaldehyde

**DOI:** 10.1107/S2056989015023658

**Published:** 2015-12-31

**Authors:** Farook Adam, Seranthimata Samshuddin, Shruthi, Badiadka Narayana, Nadiah Ameram

**Affiliations:** a School of Chemical Sciences, Universiti Sains Malaysia, 18000 Pulau Pinang, Malaysia; b Department of P.G. Studies in Chemistry, Alva’s College, Moodbidri, Karnataka 574 227, India; c Department of Studies in Chemistry, Mangalore University, Mangalagangotri, Karnataka 574 227, India

**Keywords:** crystal structure, pyrazole, hydrogen bonding

## Abstract

In the title compound, C_18_H_18_N_2_O_2_, the pyrazole ring has a twisted conformation on the CH—CH_2_ bond. The tolyl ring and the 4-meth­oxy­phenyl ring are inclined to the mean plane of the pyrazole ring by 4.40 (9) and 86.22 (9)°, respectively, while the two aromatic rings are inclined to one another by 88.75 (9)°. In the crystal, mol­ecules are linked *via* bifurcated C—H⋯(O,O) hydrogen bonds and C—H⋯π inter­actions, forming sheets lying parallel to the *ab* plane.

## Related literature   

For examples of the numerous pharmacological activities of pyrazoles, see: Samshuddin *et al.* (2012 ▸); Sarojini *et al.* (2010 ▸). For the use of 1,3,5-triaryl-2-pyrazolines as scintillation solutes, see: Wiley *et al.* (1958 ▸); and as fluorescent agents, see: Lu *et al.* (1999 ▸). For the crystal structures of pyrazoline-derived chalcones, see: Jasinski *et al.* (2012 ▸); Baktır *et al.* (2011 ▸).
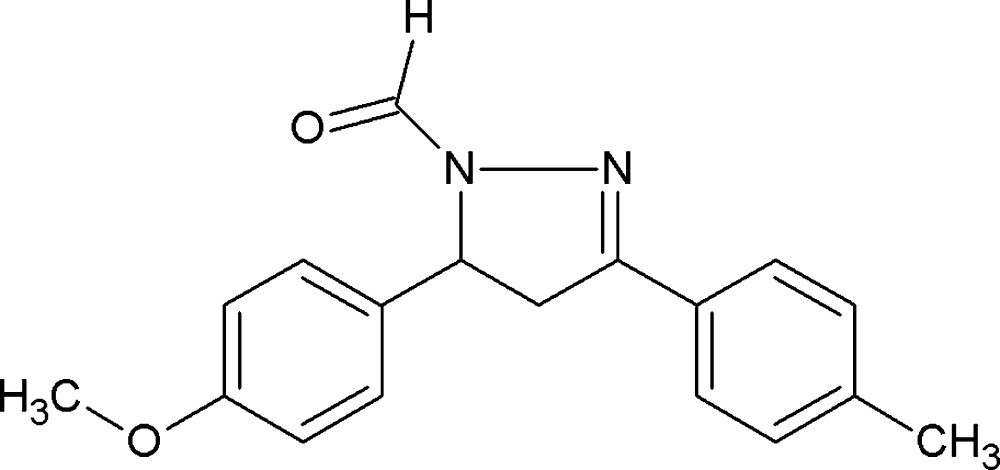


## Experimental   

### Crystal data  

C_18_H_18_N_2_O_2_
*M* *_r_* = 294.34
Monoclinic, 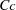

*a* = 12.0839 (9) Å
*b* = 6.4197 (5) Å
*c* = 19.7427 (18) Å
β = 104.8264 (12)°
*V* = 1480.5 (2) Å^3^
*Z* = 4
Mo *K*α radiation
μ = 0.09 mm^−1^
*T* = 100 K
0.41 × 0.23 × 0.11 mm

### Data collection  

Bruker APEXII CCD diffractometer
Absorption correction: multi-scan (*SADABS*; Bruker, 2010 ▸) *T*_min_ = 0.919, *T*_max_ = 0.965
14663 measured reflections
4301 independent reflections
4070 reflections with *I* > 2σ(*I*)
*R*_int_ = 0.023

### Refinement  

*R*[*F* ^2^ > 2σ(*F* ^2^)] = 0.036
*wR*(*F* ^2^) = 0.097
*S* = 1.05
4301 reflections
201 parameters
2 restraints
H-atom parameters constrained
Δρ_max_ = 0.28 e Å^−3^
Δρ_min_ = −0.19 e Å^−3^

### 

Data collection: *APEX2* (Bruker, 2010 ▸); cell refinement: *SAINT* (Bruker, 2010 ▸); data reduction: *SAINT*; program(s) used to solve structure: *SHELXS97* (Sheldrick 2008 ▸); program(s) used to refine structure: *SHELXL2014* (Sheldrick, 2015 ▸); molecular graphics: *SHELXTL* (Sheldrick 2008 ▸); software used to prepare material for publication: *SHELXTL*.

## Supplementary Material

Crystal structure: contains datablock(s) I, New_Global_Publ_Block. DOI: 10.1107/S2056989015023658/su5259sup1.cif

Structure factors: contains datablock(s) I. DOI: 10.1107/S2056989015023658/su5259Isup2.hkl

Supporting information file. DOI: 10.1107/S2056989015023658/su5259Isup3.cml

**Fig. 1.**
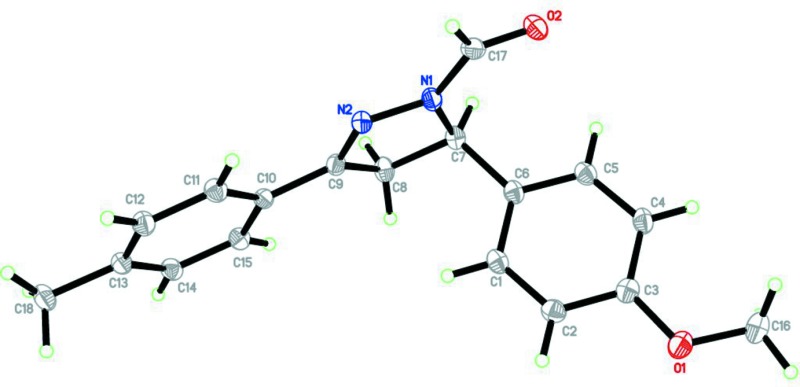
A view of the molecular structure of the title compound, with atom labelling. Displacement ellipsoids are drawn at the 50% probability level.

**Fig. 2.**
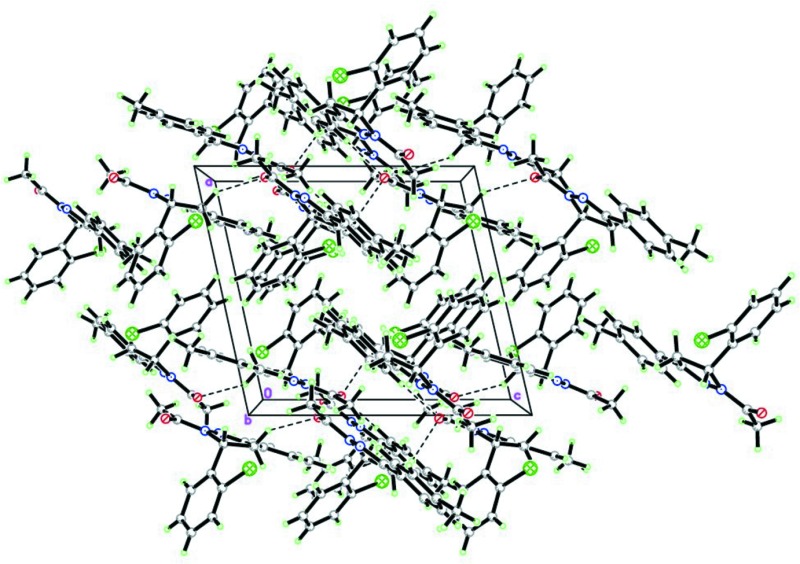
A view along the ab axis of the crystal packing of the title compound. Hydrogen bonds are shown as dashed lines (see Table 1).

. DOI: 10.1107/S2056989015023658/su5259fig1.tif
A view of the mol­ecular structure of the title compound, with atom labelling. Displacement ellipsoids are drawn at the 50% probability level.

ab . DOI: 10.1107/S2056989015023658/su5259fig2.tif
A view along the *ab* axis of the crystal packing of the title compound. Hydrogen bonds are shown as dashed lines (see Table 1).

CCDC reference: 1441491

Additional supporting information:  crystallographic information; 3D view; checkCIF report

## Figures and Tables

**Table 1:** Hydrogen-bond geometry (Å, °) *Cg*2 is the centroid of the C1–C6 ring.

*D*—H⋯*A*	*D*—H	H⋯*A*	*D*⋯*A*	*D*—H⋯*A*
C1—H1*A*⋯O2^i^	0.95	2.39	3.207 (2)	144
C14—H14*A*⋯O2^ii^	0.95	2.57	3.477 (2)	160
C15—H15*A*⋯*Cg*2^iii^	0.95	2.78	3.661 (2)	154

Symmetry codes: (i) 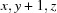; (ii) 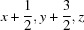; (iii) 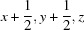.

## supplementary crystallographic information

### S1. Comment

Pyrazoline derivatives exhibit numerous pharmacological activities including anti­oxidant, anti­amoebic, anti-inflammatory, analgesic, anti­microbial, anti­depressant and anti­cancer activities (Sarojini *et al.*, 2010; Samshuddin *et al.*, 2012). Many 1,3,5-tri­aryl-2-pyrazolines have also been used as scintillation solutes (Wiley *et al.*, 1958) and as fluorescent agents (Lu *et al.*, 1999).

The crystal structures of some pyrazolines containing an *N*-alkyl chain, *viz*. 3,5-bis­(4-fluoro­phenyl)-4,5-di­hydro-1*H*-pyrazole-1 carbaldehyde (Baktır *et al.*, 2011), 3,5-bis­(4-fluoro­phenyl)-4,5-di­hydro- 1*H*-pyrazole-1-carboxamide and 3,5-bis­(4-fluoro­phenyl)-4,5-di­hydro- 1*H*-pyrazole-1-carbo­thio­amide (Jasinski *et al.*, 2012) have been reported. In view of the importance of pyrazolines, the title compound was synthesized and we report herein on its crystal structure.

The molecular structure of the title compound is illustrated in Fig. 1. The pyrazole ring has a twisted conformation on bond C7—C8. The toluyl ring and the 4-meth­oxy­phenyl ring are inclined to the mean plane of the pyrazole ring by 4.40 (9) and 86.22 (9) °, respectively. The two aromatic rings are inclined to one another by 88.75 (9) °.

In the crystal, molecules are linked *via* C—H···O hydrogen bonds and C—H···π inter­actions forming sheets lying parallel to the *ab* plane (Table 1 and Fig. 2).

### S2. Synthesis and crystallization

A mixture of (2*E*)-3-(4-meth­oxy­phenyl)-1-(4-methyl­phenyl)­prop-2-en-1-one (2.52 g, 0.01 mol) and hydrazine hydrate (1 ml) in 30 ml formic acid was refluxed for 6 h. The reaction mixture was cooled and poured into 50 ml ice-cold water. The precipitate was collected by filtration and purified by recrystallization from ethanol. Single crystals were grown from toluene by slow evaporation of the solvent (yield: 75%; m.p. 479–482 K).

### S3. Refinement

Crystal data, data collection and structure refinement details are summarized in Table 2. The hydrogen atoms were fixed geometrically (C—H = 0.95–1.00 Å) and allowed to ride on their parent atoms with *U*_iso_(H) = 1.5*U*_eq_(*C*-methyl) and 1.2*U*_eq_(C) for other H atoms.

### Crystal data

**Table:**

C_18_H_18_N_2_O_2_	*F*(000) = 624
*M**_r_* = 294.34	*D*_x_ = 1.321 Mg m^−^^3^
Monoclinic, *C**c*	Mo *K*α radiation, λ = 0.71073 Å
*a* = 12.0839 (9) Å	Cell parameters from 6705 reflections
*b* = 6.4197 (5) Å	θ = 3.5–30.2°
*c* = 19.7427 (18) Å	µ = 0.09 mm^−^^1^
β = 104.8264 (12)°	*T* = 100 K
*V* = 1480.5 (2) Å^3^	Block, colourless
*Z* = 4	0.41 × 0.23 × 0.11 mm

### Data collection

**Table:**

Bruker APEXII CCD diffractometer	4070 reflections with *I* > 2σ(*I*)
φ and ω scans	*R*_int_ = 0.023
Absorption correction: multi-scan (*SADABS*; Bruker, 2010)	θ_max_ = 30.2°, θ_min_ = 2.1°
*T*_min_ = 0.919, *T*_max_ = 0.965	*h* = −17→17
14663 measured reflections	*k* = −9→9
4301 independent reflections	*l* = −27→27

### Refinement

**Table:**

Refinement on *F*^2^	Primary atom site location: structure-invariant direct methods
Least-squares matrix: full	Secondary atom site location: difference Fourier map
*R*[*F*^2^ > 2σ(*F*^2^)] = 0.036	Hydrogen site location: inferred from neighbouring sites
*wR*(*F*^2^) = 0.097	H-atom parameters constrained
*S* = 1.05	*w* = 1/[σ^2^(*F*_o_^2^) + (0.0604*P*)^2^ + 0.3156*P*] where *P* = (*F*_o_^2^ + 2*F*_c_^2^)/3
4301 reflections	(Δ/σ)_max_ < 0.001
201 parameters	Δρ_max_ = 0.28 e Å^−^^3^
2 restraints	Δρ_min_ = −0.19 e Å^−^^3^

### Special details

**Table:**

Geometry. All e.s.d.'s (except the e.s.d. in the dihedral angle between two l.s. planes) are estimated using the full covariance matrix. The cell e.s.d.'s are taken into account individually in the estimation of e.s.d.'s in distances, angles and torsion angles; correlations between e.s.d.'s in cell parameters are only used when they are defined by crystal symmetry. An approximate (isotropic) treatment of cell e.s.d.'s is used for estimating e.s.d.'s involving l.s. planes.

### Fractional atomic coordinates and isotropic or equivalent isotropic displacement parameters (Å^2^)

**Table:**

	*x*	*y*	*z*	*U*_iso_*/*U*_eq_	
O1	0.20348 (12)	0.4331 (2)	0.35129 (8)	0.0248 (3)	
O2	0.54286 (12)	−0.1313 (2)	0.56436 (8)	0.0229 (3)	
N1	0.64240 (13)	0.1726 (2)	0.58103 (8)	0.0167 (3)	
N2	0.68881 (12)	0.3318 (2)	0.62747 (8)	0.0164 (3)	
C1	0.49214 (15)	0.4726 (3)	0.46209 (9)	0.0179 (3)	
H1A	0.5393	0.5782	0.4884	0.022*	
C2	0.38193 (16)	0.5207 (3)	0.42425 (9)	0.0189 (3)	
H2A	0.3543	0.6591	0.4246	0.023*	
C3	0.31110 (15)	0.3672 (3)	0.38556 (9)	0.0186 (3)	
C4	0.35270 (16)	0.1650 (3)	0.38360 (9)	0.0199 (4)	
H4A	0.3059	0.0603	0.3566	0.024*	
C5	0.46443 (15)	0.1188 (3)	0.42203 (9)	0.0187 (3)	
H5A	0.4930	−0.0187	0.4208	0.022*	
C6	0.53452 (15)	0.2698 (3)	0.46190 (9)	0.0168 (3)	
C7	0.65113 (15)	0.2100 (3)	0.50826 (9)	0.0171 (3)	
H7A	0.6815	0.0838	0.4894	0.021*	
C8	0.74108 (15)	0.3870 (3)	0.52205 (10)	0.0191 (3)	
H8A	0.8174	0.3345	0.5204	0.023*	
H8B	0.7182	0.5012	0.4876	0.023*	
C9	0.74102 (14)	0.4580 (3)	0.59498 (9)	0.0153 (3)	
C10	0.79896 (14)	0.6448 (2)	0.62925 (9)	0.0151 (3)	
C11	0.79596 (15)	0.6957 (3)	0.69768 (9)	0.0178 (3)	
H11A	0.7553	0.6091	0.7220	0.021*	
C12	0.85202 (15)	0.8719 (3)	0.73026 (10)	0.0191 (3)	
H12A	0.8487	0.9049	0.7766	0.023*	
C13	0.91324 (14)	1.0015 (3)	0.69604 (10)	0.0181 (3)	
C14	0.91505 (14)	0.9520 (3)	0.62759 (9)	0.0180 (3)	
H14A	0.9554	1.0394	0.6033	0.022*	
C15	0.85852 (14)	0.7760 (3)	0.59414 (9)	0.0171 (3)	
H15A	0.8604	0.7451	0.5474	0.020*	
C16	0.12863 (18)	0.2852 (4)	0.30873 (12)	0.0300 (4)	
H16A	0.0551	0.3519	0.2871	0.045*	
H16B	0.1163	0.1682	0.3378	0.045*	
H16C	0.1631	0.2340	0.2720	0.045*	
C17	0.58798 (15)	0.0120 (3)	0.60245 (10)	0.0191 (3)	
H17A	0.5844	0.0099	0.6500	0.023*	
C18	0.97566 (17)	1.1908 (3)	0.73207 (11)	0.0236 (4)	
H18A	1.0484	1.2064	0.7192	0.035*	
H18B	0.9906	1.1740	0.7829	0.035*	
H18C	0.9285	1.3151	0.7174	0.035*	

### Atomic displacement parameters (Å^2^)

**Table:**

	*U*^11^	*U*^22^	*U*^33^	*U*^12^	*U*^13^	*U*^23^
O1	0.0198 (6)	0.0266 (7)	0.0257 (7)	0.0009 (5)	0.0014 (5)	−0.0011 (5)
O2	0.0226 (6)	0.0158 (6)	0.0292 (7)	−0.0031 (5)	0.0044 (5)	−0.0006 (5)
N1	0.0171 (7)	0.0156 (6)	0.0168 (7)	−0.0029 (5)	0.0032 (5)	−0.0016 (5)
N2	0.0152 (6)	0.0153 (6)	0.0171 (7)	−0.0010 (5)	0.0015 (5)	−0.0010 (5)
C1	0.0205 (8)	0.0170 (7)	0.0171 (8)	−0.0034 (6)	0.0063 (6)	−0.0027 (6)
C2	0.0227 (8)	0.0171 (7)	0.0177 (8)	0.0006 (6)	0.0068 (6)	−0.0008 (6)
C3	0.0182 (8)	0.0226 (8)	0.0154 (7)	−0.0009 (6)	0.0050 (6)	0.0007 (6)
C4	0.0205 (9)	0.0202 (8)	0.0177 (8)	−0.0050 (6)	0.0028 (7)	−0.0028 (6)
C5	0.0213 (8)	0.0164 (7)	0.0185 (8)	−0.0016 (6)	0.0051 (7)	−0.0024 (6)
C6	0.0183 (8)	0.0171 (7)	0.0154 (8)	−0.0030 (6)	0.0051 (6)	−0.0022 (6)
C7	0.0158 (7)	0.0180 (8)	0.0176 (8)	−0.0022 (6)	0.0045 (6)	−0.0028 (6)
C8	0.0166 (7)	0.0212 (8)	0.0202 (8)	−0.0049 (6)	0.0063 (6)	−0.0034 (6)
C9	0.0131 (7)	0.0156 (7)	0.0163 (8)	−0.0002 (6)	0.0022 (6)	−0.0016 (6)
C10	0.0120 (7)	0.0139 (7)	0.0182 (8)	0.0003 (5)	0.0019 (6)	−0.0005 (6)
C11	0.0187 (8)	0.0173 (7)	0.0180 (8)	−0.0017 (6)	0.0058 (6)	0.0006 (6)
C12	0.0193 (8)	0.0186 (8)	0.0189 (8)	−0.0005 (6)	0.0040 (7)	−0.0018 (6)
C13	0.0163 (8)	0.0148 (7)	0.0216 (8)	−0.0001 (6)	0.0017 (6)	−0.0016 (6)
C14	0.0164 (8)	0.0163 (7)	0.0217 (8)	−0.0016 (6)	0.0055 (7)	0.0005 (6)
C15	0.0165 (7)	0.0173 (7)	0.0180 (8)	−0.0009 (6)	0.0057 (6)	−0.0001 (6)
C16	0.0212 (9)	0.0341 (10)	0.0294 (11)	−0.0026 (8)	−0.0031 (8)	−0.0018 (8)
C17	0.0172 (8)	0.0163 (7)	0.0233 (9)	0.0007 (6)	0.0043 (7)	0.0036 (6)
C18	0.0242 (9)	0.0168 (8)	0.0280 (10)	−0.0041 (6)	0.0035 (7)	−0.0046 (7)

### Geometric parameters (Å, º)

**Table:**

O1—C3	1.370 (2)	C8—H8A	0.9900
O1—C16	1.427 (2)	C8—H8B	0.9900
O2—C17	1.225 (2)	C9—C10	1.464 (2)
N1—C17	1.348 (2)	C10—C11	1.399 (2)
N1—N2	1.3916 (19)	C10—C15	1.401 (2)
N1—C7	1.487 (2)	C11—C12	1.389 (2)
N2—C9	1.294 (2)	C11—H11A	0.9500
C1—C2	1.385 (3)	C12—C13	1.397 (2)
C1—C6	1.400 (2)	C12—H12A	0.9500
C1—H1A	0.9500	C13—C14	1.394 (3)
C2—C3	1.397 (2)	C13—C18	1.509 (2)
C2—H2A	0.9500	C14—C15	1.396 (2)
C3—C4	1.396 (2)	C14—H14A	0.9500
C4—C5	1.401 (3)	C15—H15A	0.9500
C4—H4A	0.9500	C16—H16A	0.9800
C5—C6	1.391 (2)	C16—H16B	0.9800
C5—H5A	0.9500	C16—H16C	0.9800
C6—C7	1.520 (2)	C17—H17A	0.9500
C7—C8	1.547 (2)	C18—H18A	0.9800
C7—H7A	1.0000	C18—H18B	0.9800
C8—C9	1.510 (2)	C18—H18C	0.9800
			
C3—O1—C16	117.61 (16)	N2—C9—C8	113.76 (14)
C17—N1—N2	120.11 (15)	C10—C9—C8	124.81 (15)
C17—N1—C7	126.02 (15)	C11—C10—C15	118.75 (15)
N2—N1—C7	113.67 (13)	C11—C10—C9	120.65 (15)
C9—N2—N1	107.38 (14)	C15—C10—C9	120.60 (16)
C2—C1—C6	120.53 (16)	C12—C11—C10	120.46 (16)
C2—C1—H1A	119.7	C12—C11—H11A	119.8
C6—C1—H1A	119.7	C10—C11—H11A	119.8
C1—C2—C3	120.51 (16)	C11—C12—C13	121.13 (16)
C1—C2—H2A	119.7	C11—C12—H12A	119.4
C3—C2—H2A	119.7	C13—C12—H12A	119.4
O1—C3—C4	125.21 (16)	C14—C13—C12	118.34 (16)
O1—C3—C2	115.02 (16)	C14—C13—C18	120.72 (16)
C4—C3—C2	119.76 (16)	C12—C13—C18	120.95 (17)
C3—C4—C5	119.09 (16)	C13—C14—C15	121.08 (16)
C3—C4—H4A	120.5	C13—C14—H14A	119.5
C5—C4—H4A	120.5	C15—C14—H14A	119.5
C6—C5—C4	121.44 (16)	C14—C15—C10	120.23 (16)
C6—C5—H5A	119.3	C14—C15—H15A	119.9
C4—C5—H5A	119.3	C10—C15—H15A	119.9
C5—C6—C1	118.65 (16)	O1—C16—H16A	109.5
C5—C6—C7	120.08 (15)	O1—C16—H16B	109.5
C1—C6—C7	121.11 (15)	H16A—C16—H16B	109.5
N1—C7—C6	109.79 (14)	O1—C16—H16C	109.5
N1—C7—C8	99.73 (14)	H16A—C16—H16C	109.5
C6—C7—C8	114.96 (15)	H16B—C16—H16C	109.5
N1—C7—H7A	110.6	O2—C17—N1	123.94 (18)
C6—C7—H7A	110.6	O2—C17—H17A	118.0
C8—C7—H7A	110.6	N1—C17—H17A	118.0
C9—C8—C7	102.57 (14)	C13—C18—H18A	109.5
C9—C8—H8A	111.3	C13—C18—H18B	109.5
C7—C8—H8A	111.3	H18A—C18—H18B	109.5
C9—C8—H8B	111.3	C13—C18—H18C	109.5
C7—C8—H8B	111.3	H18A—C18—H18C	109.5
H8A—C8—H8B	109.2	H18B—C18—H18C	109.5
N2—C9—C10	121.34 (15)		
			
C17—N1—N2—C9	176.47 (16)	N1—C7—C8—C9	−15.61 (17)
C7—N1—N2—C9	−8.36 (18)	C6—C7—C8—C9	101.70 (17)
C6—C1—C2—C3	0.3 (3)	N1—N2—C9—C10	179.55 (14)
C16—O1—C3—C4	−1.7 (3)	N1—N2—C9—C8	−3.61 (19)
C16—O1—C3—C2	177.67 (17)	C7—C8—C9—N2	13.1 (2)
C1—C2—C3—O1	179.00 (16)	C7—C8—C9—C10	−170.18 (15)
C1—C2—C3—C4	−1.6 (3)	N2—C9—C10—C11	−2.2 (2)
O1—C3—C4—C5	−179.20 (17)	C8—C9—C10—C11	−178.69 (17)
C2—C3—C4—C5	1.5 (3)	N2—C9—C10—C15	177.74 (16)
C3—C4—C5—C6	−0.1 (3)	C8—C9—C10—C15	1.3 (2)
C4—C5—C6—C1	−1.2 (3)	C15—C10—C11—C12	−0.6 (2)
C4—C5—C6—C7	174.34 (16)	C9—C10—C11—C12	179.34 (16)
C2—C1—C6—C5	1.1 (3)	C10—C11—C12—C13	−0.5 (3)
C2—C1—C6—C7	−174.42 (16)	C11—C12—C13—C14	1.2 (3)
C17—N1—C7—C6	69.3 (2)	C11—C12—C13—C18	−178.97 (17)
N2—N1—C7—C6	−105.52 (16)	C12—C13—C14—C15	−0.8 (3)
C17—N1—C7—C8	−169.58 (16)	C18—C13—C14—C15	179.34 (16)
N2—N1—C7—C8	15.59 (18)	C13—C14—C15—C10	−0.3 (3)
C5—C6—C7—N1	−95.70 (18)	C11—C10—C15—C14	1.0 (2)
C1—C6—C7—N1	79.7 (2)	C9—C10—C15—C14	−178.97 (15)
C5—C6—C7—C8	152.86 (16)	N2—N1—C17—O2	178.37 (16)
C1—C6—C7—C8	−31.7 (2)	C7—N1—C17—O2	3.8 (3)

### Hydrogen-bond geometry (Å, º)

Cg2 is the centroid of the C1–C6 ring.

**Table:**

*D*—H···*A*	*D*—H	H···*A*	*D*···*A*	*D*—H···*A*
C1—H1*A*···O2^i^	0.95	2.39	3.207 (2)	144
C14—H14*A*···O2^ii^	0.95	2.57	3.477 (2)	160
C15—H15*A*···*Cg*2^iii^	0.95	2.78	3.661 (2)	154

Symmetry codes: (i) *x*, *y*+1, *z*; (ii) *x*+1/2, *y*+3/2, *z*; (iii) *x*+1/2, *y*+1/2, *z*.
