# Supplementary figures and images for: Crystal structure of 5-(4-meth­oxy­phen­yl)-3-(4-methyl­phen­yl)-4,5-di­hydro-1H-pyrazole-1-carbaldehyde
Source: Acta Crystallogr E Crystallogr Commun. 2015 Dec 31;71(Pt 12):o1093–4. doi: 10.1107/S2056989015023658 (PMC4719996; doi:10.1107/S2056989015023658)

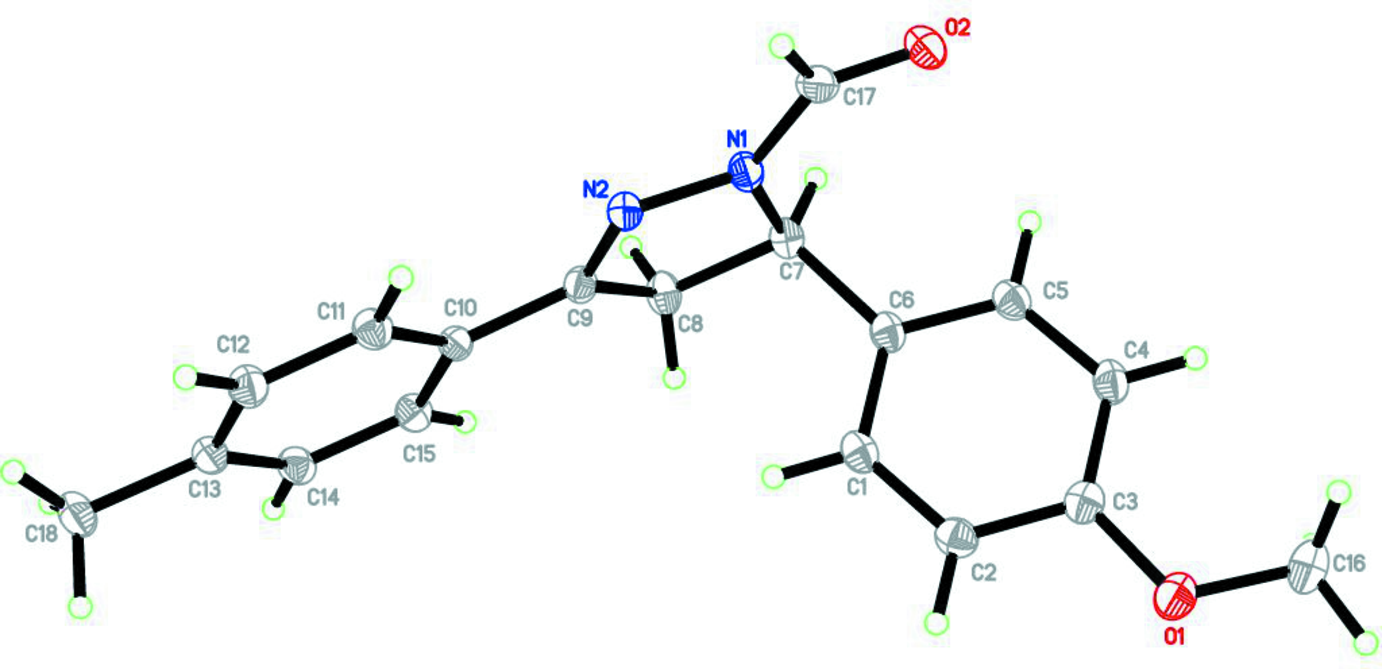

Supplement: Supplementary file 4 [file e-71-o1093-fig1.tif]

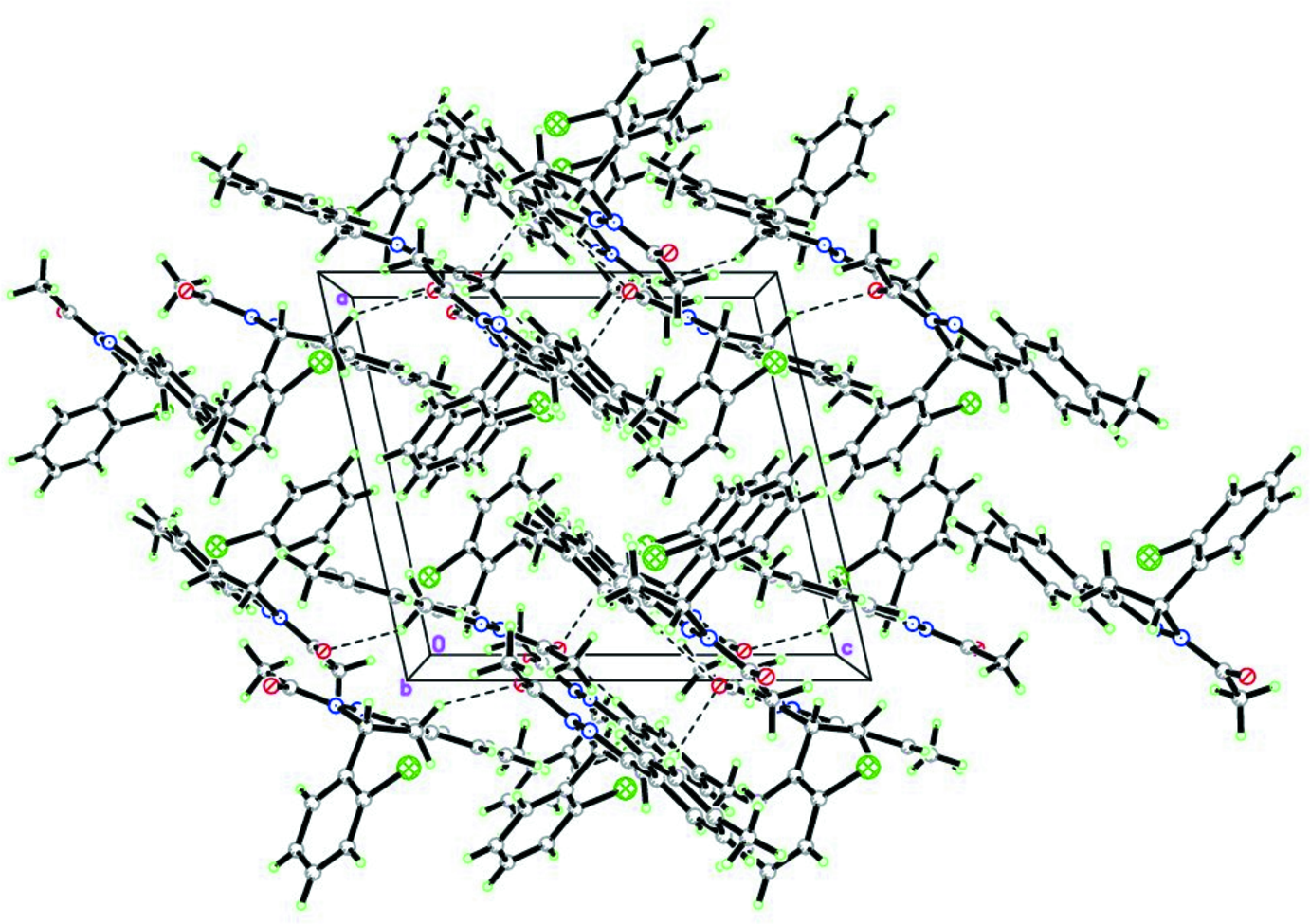

Supplement: Supplementary file 5 [file e-71-o1093-fig2.tif]
